# Maury's Tooth Brushes

**Published:** 1869-02

**Authors:** 


*Maury's Tooth Brushes.*—We have recently been much pleased with an improved tooth-brush invented by T. F. Maury.

The improvement consists in the tapering and polishing of the ends of the bristles, all sharp, angular extremities being removed, so that the points enter between the approximal surfaces of the teeth and very effectually remove extraneous substances. This improvement in the shape of the free extremities of the bristles also causes the brush to glide more smoothly over the gums thereby preventing irritation and the recession of the gums from the neck of the teeth.

Lilly & Brother 24 S. Sharp St., Baltimore. are the agents for this and the adjoining States.

